# Variations in Central Adiposity, Cardiovascular Fitness, and Objectively Measured Physical Activity According to Weight Status in Children (9–11 Years)

**DOI:** 10.3389/fphys.2019.00936

**Published:** 2019-07-24

**Authors:** Mustafa Söğüt, Filipe Manuel Clemente, Cain C. T. Clark, Pantelis Theodoros Nikolaidis, Thomas Rosemann, Beat Knechtle

**Affiliations:** ^1^Faculty of Sport Sciences, Kırıkkale University, Kırıkkale, Turkey; ^2^School of Sport and Leisure, Polytechnic Institute of Viana do Castelo, Melgaço, Portugal; ^3^Instituto de Telecomunicações, Delegação da Covilhã, Covilhã, Portugal; ^4^Centre for Sport, Exercise and Life Sciences, Coventry University, Coventry, United Kingdom; ^5^Exercise Physiology Laboratory, Nikaia, Greece; ^6^Institute of Primary Care, University of Zurich, Zurich, Switzerland; ^7^Medbase St. Gallen Am Vadianplatz, St. Gallen, Switzerland

**Keywords:** physical activity, adiposity, cardiovascular fitness, accelerometry, BMI

## Abstract

The purpose of this study was twofold: first, to compare the central adiposity (CA), cardiovascular fitness (CF), and physical activity (PA) in children with different weight status, and second, to determine the associations between moderate to vigorous physical activity (MVPA) and measures of adiposity [CA and body mass index (BMI)] and CF. A sample of 244 children (boys = 120 and girls = 124), 9.7–10.8 years of age (10.3 ± 0.3 years), was measured for stature, body mass, waist circumferences, and 20-m multi-stage fitness test. PA was recorded with ankle mounted accelerometer. BMI groups were used to classify children as underweight (UW), normal weight (NW), and overweight (OW). The prevalence of being OW was 21.7 and 25% in boys and girls, respectively. Only 5.3% of the participants were found to accumulate recommended amount (≥60 min/day) of MVPA. Boys were significantly outperformed girls in terms of CF. Moreover, they were significantly more engaged in moderate and vigorous physical activities than girls. Regardless of gender, results indicated that OW children had significantly higher values in all anthropometric parameters and lower level of CF than their UW and NW counterparts. In girls, OW children were found to accrue less time engaging in MVPA than the children in UW and NW groups. In boys, OW children were found to accrue less time engaging in vigorous activities than UW and NW children. Results also showed that there were no significant differences between UW and NW girls and boys in respect to CF. Besides, UW girls were found to accrue more time engaging in MVPA than NW girls. MVPA was found to be significantly and negatively correlated with BMI and waist circumference and significantly and positively correlated with CF in both boys and girls. These discrepancies and associations highlight the considerable influences of MVPA on weight status and CF in children.

## Introduction

The progressive increase in childhood obesity is believed to result in pathophysiological consequences, including incidence of cardiovascular events in adulthood (Ayer et al., 2015; McCrindle, 2015). For that reason, among many, an early detection of adverse cardio-metabolic risk profiles may promote a beneficial effect to prevent or protect against future morbidity by adopting specific intervention programs that may help to reduce the risk factors (Bailey et al., 2015). Among other factors, an increase in physical activity (PA) levels, systematization, quality, and intensity can be a safe and effective contributor to ameliorate the risk of morbidity, concomitantly increasing the quality of life of the citizens (Clark et al., 2016; Higuera-Hernández et al., 2018).

Empirical evidence is persistently supportive of the assertion that more PA accrued, the greater the health benefits, particularly of moderate-to-vigorous intensity (Janssen and LeBlanc, 2010). However, it is commonly observed that overweight (OW) children are typically less active than their normal-weight counterparts (Stratton et al., 2007). Moreover, a lack of motor competency also putatively mediates low PA levels; thus, PA programs that promote an improvement in fundamental movement skills may facilitate improvement of PA levels (Utesch et al., 2018). In fact, it seems that a highly developed level of motor competency in childhood may have the potential to foster lifelong functional independence and quality of life (Robinson et al., 2015; Clark et al., 2018).

One of the capacities typically included in batteries of motor competence assessment is aerobic cardiovascular fitness (CF; Khodaverdi et al., 2016; Luz et al., 2019). Cardiovascular capacity seems to play an important role in sustaining a healthier cardiovascular profile in young children and adolescents (Ruiz et al., 2015). Moreover, aerobic fitness is closely related to PA levels. In fact, in two cross-sectional studies, it was found that children with high moderate to vigorous physical activity (MVPA) levels and low sedentary behaviors had higher odds of having a high CF (Martinez-Gomez et al., 2011; Santos et al., 2014).

Briefly, the evidence suggests that less active children presents higher odds of having excess weight and lower CF. Such hypotheses should be better analyzed, namely because they can serve as a “red flag” to control and implement monitoring strategies in the early stages of childhood. Previous studies have widely investigated the PA, aerobic fitness, and adiposity of normal weight (NW) and OW children. However, there is paucity of information for children who are underweight (UW). Thus, in this study, we examined whether standardized measure of PA, central adiposity (CA), and CF were different in UW, NW, and OW children in both genders. Furthermore, we analyze the associations between MVPA and markers of adiposity [CA and body mass index (BMI)] and CF.

## Materials and Methods

### Participants

A total of 244 children (boys = 120 and girls = 124), 9.7–10.8 years of age (10.3 ± 0.3 years), participated in the study. They were a voluntary sub-sample of 822 children from 30 publicly funded primary schools in Southern Wales, UK. Prior to research commencing, legal guardian informed consent and child assent were attained. This research was conducted in agreement with the guidelines and policies of the institutional ethics committee and in accordance with the Declaration of Helsinki.

### Anthropometric Measurements

Measurements were taken by a single observer in accordance with standardized procedures (Lohman et al., 1988). Stature was measured with a stadiometer (SECA, Hamburg, Germany) to the nearest 0.01 m. Body mass was measured with a digital scale (SECA, Hamburg, Germany) to the nearest 0.1 kg. Waist circumference was measured with a flexible steel tape to the nearest centimeter and at the smallest circumference between the ribs and iliac crest. BMI was calculated by dividing weight (kg) by the squared height (m). BMI centiles were used to classify children as either UW (<5th percentile), NW (5th to 85th percentile), and OW (>85th to <95th percentile) (Cole and Lobstein, 2012).

### Twenty-Meter Multi-Stage Fitness Test

Multi-stage fitness test (MSFT) was used to assess CF of the participants. All participants were familiar with the MSFT procedures, since they have routinely been involved in this test at school. Nevertheless, instructions were provided, verbatim, prior to the commencement of the test, according to the standardized guidance of Leger et al. (1988). Moreover, the test was conducted on an indoor training facility (50 m × 50 m) in Southern Wales (UK). The participants completed the MSFT by running back and forth along a 20 m course and were required to touch the 20 m line at the same time where a sound signal was emitted from a pre-recorded audio disk. The frequency of the sound emissions increased to produce a corresponding increase in running speed. The test stopped when the participant reached volitional exhaustion and was no longer able to follow the set pace, or participants were withdrawn after receiving two verbal warnings to meet the required pace (Leger et al., 1988).

### Physical Activity

Study participants wore an ActiGraph GT3X+ accelerometer (ActiGraph, Pensacola, FL, USA) for 7 consecutive days in summer (Fuemmeler et al., 2011; Ridgers et al., 2014). Participants were instructed to wear the accelerometer constantly except when bathing or swimming. The accelerometer dimensions are 4.6 cm × 3.3 cm × 1.5 cm and weigh 19 g. Its sampling frequency was set to 100 Hz, and the sampling interval (epoch) in the present study was set to be 1–s (Pate et al., 2006; Østbye et al., 2013). Participants wore their accelerometer on the waist, above the right hip, affixed using an elastic belt (Hesketh et al., 2014). Accelerometer data were analyzed to measure the following parameters: daily duration of sedentary behavior, light PA, moderate PA, vigorous PA, and MVPA (Migueles et al., 2017). ActiGraph acceleration data were analyzed using a commercially available analysis tool (KineSoft version 3.3.67, KineSoft; www.kinesoft.org). Non-wear periods were defined as any sequence of >20 consecutive minutes of zero activity counts (Tudor-Locke et al., 2015). Sedentary behavior was defined as <100 counts per minute, while 100, 2,296, and 4,012 counts per minute were thresholds to define light, moderate, and vigorous PA, respectively (Evenson et al., 2008; Trost et al., 2011). All PA data were collected in week blocks, over a 1-month period.

### Statistical Procedures

All data were analyzed using SPSS for Windows. Descriptive statistics (mean ± SD) were calculated for the variables. Due to non-normal distribution of the data, the Kruskal-Wallis test was used to analyze the discrepancies of variables in regard to BMI categories. Mann-Whitney *U* tests were used to follow-up pairwise comparisons and to examine gender differences. Spearman’s rank correlation coefficient was conducted to examine the association between MVPA and other variables. Statistical significance level was settled at 0.05.

## Results

Descriptive statistics are detailed in Table 1. The results revealed that boys were significantly (*p* < 0.05) outperformed girls in terms of CF. Furthermore, they were significantly (*p* < 0.01) more engaged in moderate and vigorous physical activities than girls. Other variables were found comparable between genders.

**Table 1:** Descriptive statistics of boys and girls and the Mann-Whitney *U* test results.

Variables	Male (*n* = 120)	Female (*n* = 124)	*U*	*p*
Age (years)	10.28 ± 0.30	10.27 ± 0.31	7.2	0.697
Height (cm)	140.7 ± 5.7	141.8 ± 6.6	6.9	0.294
Body mass (kg)	36.1 ± 8.7	38.6 ± 10.6	6601.0	0.128
BMI (kg/m^2^)	18.1 ± 3.3	18.9 ± 3.9	6.6	0.138
Waist circumference (cm)	64.1 ± 10.0	65.2 ± 10.0	7.4	0.914
20 m shuttle (laps)	31.6 ± 16.7	26.6 ± 12.2	6.3	0.039
Wear time (min/day)	1413.5 ± 49.8	1396.7 ± 72.9	6719.0	0.180
Sedentary time (min/day)	510.2 ± 80.7	503.1 ± 63.8	7306.0	0.808
Light activity (min/day)	351.8 ± 61.5	367.1 ± 55.6	6.6	0.133
Moderate activity (min/day)	20.9 ± 9.9	15.7 ± 7.0	5059.5	0.001
Vigorous activity (min/day)	15.1 ± 9.0	9.3 ± 5.7	4.5	0.001
MVPA (min/day)	36.1 ± 18.5	25.0 ± 12.3	4.8	0.001
Sleep time (min/day)	541.9 ± 31.9	544.7 ± 39.1	6.6	0.114

The descriptive statistics of boys by weight status and the Kruskal-Wallis test results are given in Table 2. The Mann-Whitney *U* test revealed that OW boys had significantly higher values in all anthropometric parameters than their UW and NW counterparts. Further results indicated that OW boys had significantly lower level of CF than boys in other BMI groups. Moreover, they were found to accrue less time engaging in vigorous activities than UW and NW boys. There were no significant differences between UW and NW boys in respect to CF and PA variables.

**Table 2:** Descriptive statistics of boys by weight status and the Kruskal-Wallis test results.

BMI categories	UW (*n* = 12)	NW (*n* = 82)	OW (*n* = 26)	*H*	*p*
Age (years)	10.03 ± 0.31	10.30 ± 0.28	10.31 ± 0.30	8.8	0.012
Height (cm)	135.6 ± 4.1	139.9 ± 4.7	145.2 ± 6.6	23.7	0.001
Body mass (kg)	25.6 ± 2.8	33.5 ± 3.8	49.2 ± 7.5	73.5	0.001
BMI (kg/m^2^)	13.9 ± 1.2	17.1 ± 1.5	23.2 ± 2.0	79.5	0.001
Waist circumference (cm)	56.7 ± 2.5	60.7 ± 6.9	78.2 ± 7.2	67.8	0.001
20 m shuttle (laps)	39.1 ± 22.8	34.1 ± 15.8	20.3 ± 10.6	17.7	0.001
Wear time (min/day)	1408.5 ± 45.8	1415.4 ± 44.8	1409.8 ± 65.9	0.4	0.829
Sedentary time (min/day)	485.2 ± 70.6	506.0 ± 74.6	534.9 ± 98.8	3.7	0.156
Light activity (min/day)	370.5 ± 65.2	355.5 ± 57.5	331.3 ± 69.2	4.5	0.106
Moderate activity (min/day)	21.4 ± 10.8	22.3 ± 8.9	16.6 ± 11.7	12.5	0.002
Vigorous activity (min/day)	17.8 ± 9.7	16.4 ± 8.8	9.9 ± 7.4	14.9	0.001
MVPA (min/day)	39.2 ± 20.5	38.7 ± 17.2	26.5 ± 18.9	14.1	0.001
Sleep time (min/day)	545.1 ± 38.9	539.8 ± 31.7	547.3 ± 29.4	0.607	1.5

The descriptive statistics of girls by weight status and the Kruskal-Wallis test results are presented in Table 3. The Mann-Whitney *U* test demonstrated that OW girls had significantly greater values in all anthropometric parameters than their UW and NW counterparts. Furthermore, they were significantly less engaged in moderate and vigorous physical activities than UW and NW girls. Besides, they were found to have significantly lower level of CF than the girls in other BMI groups. UW girls were found to accrue more time engaging in moderate and vigorous physical activities than NW girls. There were no significant differences between UW and NW girls in terms of CF.

**Table 3:** Descriptive statistics of girls by weight status and the Kruskal-Wallis test results.

BMI categories	UW (*n* = 10)	NW (*n* = 83)	OW (*n* = 31)	*H*	*p*
Age (years)	10.04 ± 0.18	10.26 ± 0.32	10.35 ± 0.30	7.4	0.024
Height (cm)	137.0 ± 3.5	140.6 ± 5.9	146.5 ± 6.6	25.6	0.001
Body mass (kg)	26.6 ± 1.1	34.4 ± 4.8	53.6 ± 8.4	79.0	0.001
BMI (kg/m^2^)	14.2 ± 0.3	17.3 ± 1.5	24.9 ± 2.8	84.1	0.001
Waist circumference (cm)	54.9 ± 2.6	61.3 ± 5.6	78.9 ± 7.2	70.5	0.001
20 m shuttle (laps)	33.8 ± 11.3	30.3 ± 11.0	14.4 ± 5.6	48.8	0.001
Wear time (min/day)	1370.4 ± 140.5	1399.8 ± 64.9	1397.0 ± 63.6	2.1	0.357
Sedentary time (min/day)	480.4 ± 70.9	505.9 ± 66.6	503.2 ± 53.6	0.4	0.803
Light activity (min/day)	391.2 ± 62.9	365.3 ± 57.2	364.4 ± 48.3	1.5	0.465
Moderate activity (min/day)	22.3 ± 9.1	16.0 ± 6.6	12.5 ± 5.7	12.8	0.002
Vigorous activity (min/day)	14.3 ± 5.9	9.8 ± 5.7	6.6 ± 3.8	19.0	0.001
MVPA (min/day)	36.6 ± 14.7	25.8 ± 11.9	19.1 ± 9.1	15.8	0.001
Sleep time (min/day)	531.9 ± 75.0	543.1 ± 37.5	553.4 ± 24.7	1.1	0.587

The correlations between MVPA and the other variables were presented separately for each gender in Table 4. The results showed that MVPAs were significantly and negatively associated with BMI and waist circumference and significantly and positively associated with CF in both boys and girls.

**Table 4:** Correlation results between MVPA and other variables by gender.

Variables	Boys		Girls	
	*r*_s_	*p*	*r*_s_	*p*
BMI	−0.230	0.012	−0.333	0.001
Waist circumference	−0.179	0.049	−0.292	0.001
20 m shuttle run	0.490	0.001	0.236	0.008

## Discussion

The main purpose of this cross-sectional study was to determine the variations in the CA, CF, and PA among children with different weight status. The results indicated that regardless of gender, OW children were found to have greater CA and lower values in CF and MVPA than their UW and NW counterparts. These findings are in line with the results of previous investigations, where significant differences among BMI groups have been reported in regard to CA (Ferreira and Marques-Vidal, 2008; Karppanen et al., 2012), CF (Aires et al., 2010; Niederer et al., 2012), and MVPA (Page et al., 2005; Decelis et al., 2014). Although current literature has not reached a consensus regarding the actual causes of child OW/obesity, such disparities among children with different weight status may be, at least in part, attributed to the unhealthy eating habits, physical inactivity, sedentary screen time, environmental factors, and psychological stress (Slyper, 2004; Pate et al., 2013; Ross et al., 2016).

The results showed that, regardless of gender, there were no significant differences between UW and NW children in respect to CF. This result is in accord with the finding of the earlier examinations (Artero et al., 2010; Gulías-González et al., 2014). On the other hand, UW girls were found to accrue more time engaging in moderate and vigorous physical activities than NW girls. Previous studies present inconsistent results in regard to PA level of UW and NW children (Chung et al., 2012; Fairclough et al., 2017). Therefore, further research is needed on this important subject.

The results in regard to gender effect demonstrated that boys scored significantly better than girls in terms of CF. In addition, they were found to be significantly more engaged in MVPA than girls. These results are in accord with the findings of antecedent studies (Hussey et al., 2007; Pereira et al., 2010; Ridgers et al., 2010; Martinez-Gomez et al., 2011). This difference in PA level was previously explained by social support (Edwardson et al., 2013), perceived enjoyment of physical education (Cairney et al., 2012), and biological maturation (Wickel et al., 2009). Gender differences in CF may be due to the disparity in body mass, where girls were found to be significantly heavier than boys (Armstrong and Welsman, 1994; Martinez-Gomez et al., 2011).

The results of the correlation analysis demonstrated small to medium associations between MVPA and BMI, waist circumference, and aerobic fitness in both girls and boys. Similar observations were reported with the previous studies (Gutin et al., 2005; Ruiz et al., 2006; Jiménez-Pavón et al., 2010). It seems that participating in MVPA plays not only an important role on having a healthy central and total adiposity but also on aerobic fitness in childhood.

The overall prevalence of OW was found to be 25.4% (boys = 21.7% and girls = 25.0%). These percentages were higher than the previously measured children of similar age; for example, Chinn and Rona (2001) studied the secular tends in OW among British children aged between 9 and 11 years. In boys, prevalence of OW in 1974, 1984, and 1994 was reported as 6.2, 5.8, and 12.7, respectively. In girls, it was noted as 9.9, 9.9, and 16.7, respectively. Correspondingly, lower level of OW prevalence was observed for the children living in Wales (girls = 15.8% and boys = 17.9%) (Elgar et al., 2005) and living in Ireland (girls = 20.7% and boys = 20.2; Hussey et al., 2007). Additionally, in a recent paper (Skinner et al., 2018), lower prevalence of being OW (18.5%) was found in US children (9–11 years). This result might be due to that only 5.3% of the participants were found to accumulate recommended amount (≥60 min/day) of MVPA (World Health Organization, 2010). In summary, these findings undoubtedly indicate the existence of excessive adiposity in children.

## Limitations

It should be acknowledged that the present study has several limitations. First, the cross-sectional observational design employed in this study precludes cause and effect interpretations. Second, biological maturity status, one of the major potential confounding factors for physical and functional characteristics of children in these age groups, was not evaluated. Third, in addition to BMI, estimation of body fat percentage would help to improve the interpretation of data in regard to body composition. Fourth, weather-related variables (temperature, rain, wind, etc.) were not evaluated. Lastly, rather than fractionation (intermittent versus continuous), only cumulative amounts of MVPA were considered in this study.

## Conclusions

In this study, we examined whether children with different weight status differ in respect to their PA, CA, and CF. It is evident that, regardless of gender, OW children had significantly lower CF than their UW and NW counterparts. In girls and boys, OW children accrued less MVPA than the children in UW and NW groups. This study is original in the sense that it also provides data not only for NW and OW children but also for their UW counterparts. Our findings indicated that there were no significant differences between UW and NW girls and boys in terms of CF. Moreover, UW girls were found to accrue more time engaging in moderate and vigorous physical activities than NW girls. MVPA was found to be significantly and negatively correlated with BMI and waist circumference and significantly and positively correlated with CF in both boys and girls. These discrepancies and associations highlight the considerable influences of MVPA on weight status and CF in children, highlighting the need for sustained and longitudinal monitoring, in addition to greater effort given employing interventions, relevant to PA and CF.

## Data Availability

The datasets generated for this study are available on request to the corresponding author.

## Ethics Statement

Ethical approval prior to research commencing, legal guardian informed consent and child assent were attained. This research was conducted in agreement with the guidelines and policies of the institutional ethics committee and in accordance with the Declaration of Helsinki. The article was approved by a local ethical committee with the number IPVC-ESDL13092018.

## Author Contributions

MS, FC, and CC designed the study. CC collected the data. MS analyzed and interpreted the data and drafted the manuscript. PN, TR, and BK critically revised the paper. All authors discussed the results and contributed to the final version of the article.

### Conflict of Interest Statement

The authors declare that the research was conducted in the absence of any commercial or financial relationships that could be construed as a potential conflict of interest.
